# Supplementary material for: Ferroptosis, Inflammation, and Microbiome Alterations in the Intestine in the Göttingen Minipig Model of Hematopoietic-Acute Radiation Syndrome
Source: Int J Mol Sci. 2024 Apr 20;25(8):4535. doi: 10.3390/ijms25084535 (PMC11050692; doi:10.3390/ijms25084535)
Supplement: Supplementary file 1 [file ijms-25-04535-s001.zip › ijms-2928198-supplementary.pdf]

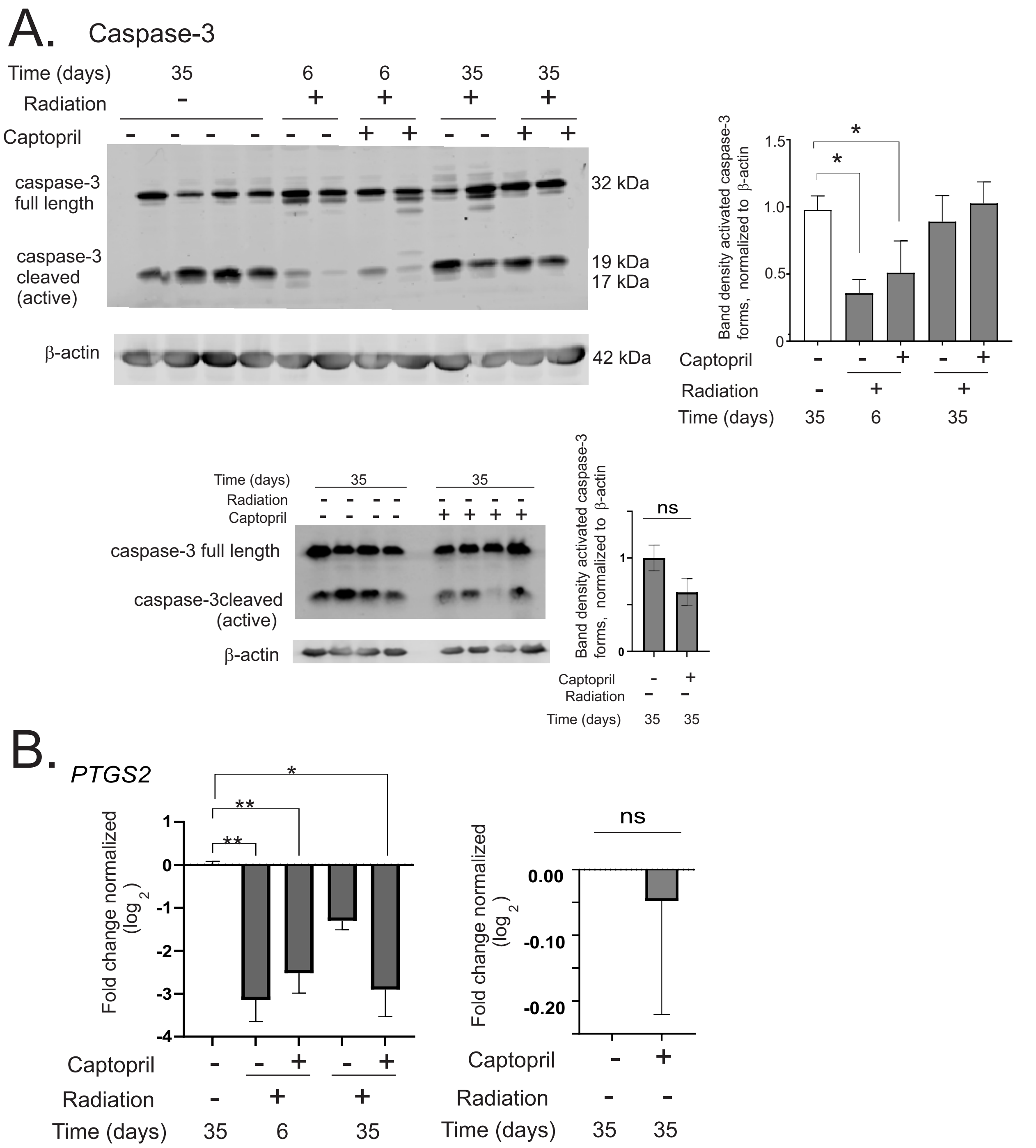

Figure S1. Effects of captopril on radiation-induced changes in caspase-3 activation and PTGS2 regulation. Göttingen minipgs, 4-6 months of age, were sham irradiated or exposed to TBI. Minipigs received either vehicle or captopril twice daily starting 4 h post-irradiation through 12 days post-irradiation. Tissues were obtained 6 or 35 days post-irradiation. A. Western blot of caspase-3 full length (inactive) and cleaved (activated). Representative data are shown from n = 4 animals per group. Bar graph shows means of band density +/- SEM of activated caspase-3 normalized to β-actin. B. qPCR of *PTGS2* gene expression (log2 relative expression) normalized to GAPDH. Graph shows means +/- SEM, n=4 animals per group. For all graphs, ns indicates not significant, * indicates $p < 0.05$; ** indicates $p < 0.01$.

Firmicutes/Bacteriodetes

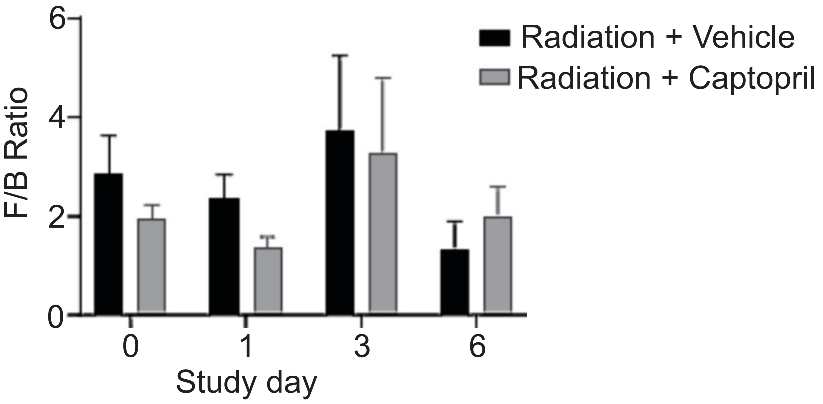

Figure S2. Effects of captopril on radiation-induced changes in the Firmicutes/ Bacteriodetes ratio. Göttingen minipgs, 4-6 months of age, were sham irradiated or exposed to TBI. Minipigs received either vehicle (yogurt) or captopril twice daily starting 4 h post-irradiation through 12 days post-irradiation. Tissues were obtained 6 or 35 days post-irradiation. The ratio of Firmicutes/Bacteriodetes phyla were calculated for each study day. Data show means +/- SEM, n=4/group. No significance was found for any time point or group compared with the pre-irradiation time (0).

A.

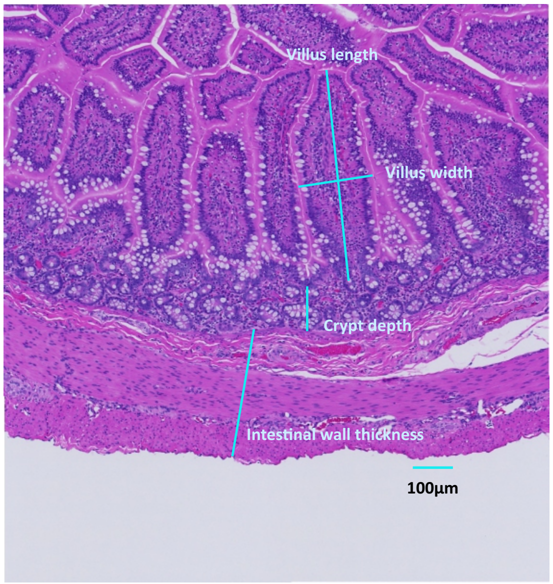

B.

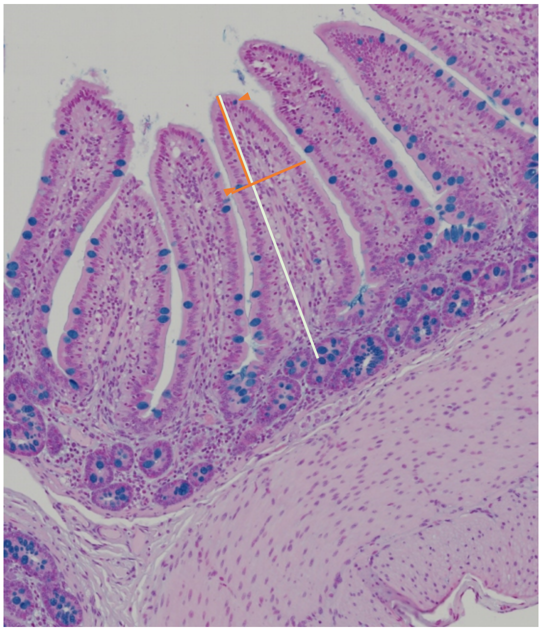

Figure S3. Schematics of histological measurements in Zen. Representative images of intestine histology, 20x images are shown. (**A**) Lines were drawn in Zen software to mark the villus length, villus width, crypt depth and intestinal wall thickness. Lines indicate how measurements were made in the software. Metadata collected in Zen yields the micron measurements. (**B**) Goblet cell localization in the top 1/3 of the villus was quantified. The white line indicates the measurement taken for the length of the villus with the orange line indicating 1/3 distance from the tip. All measurements were performed by a trained researcher blinded to the treatment groups.
